# Food and Nutrients Intake in the School Lunch Program among School Children in Shanghai, China

**DOI:** 10.3390/nu9060582

**Published:** 2017-06-07

**Authors:** Zhenru Huang, Runying Gao, Nadila Bawuerjiang, Yali Zhang, Xiaoxu Huang, Meiqin Cai

**Affiliations:** School of Public Health, Shanghai Jiao Tong University,227 South Chongqing Rd, Huangpu Qu, Shanghai 200025, China; hzhenru@163.com (Z.H.); dly-slyj@hotmail.com (R.G.); nadirah@126.com (N.B.); abeney@126.com (Y.Z.); xiaoxuhuang0909@126.com (X.H.)

**Keywords:** students, school lunch, nutrient, intake, plate waste

## Abstract

This study aimed to evaluate the intake of food and nutrients among primary, middle, and high schools students in Shanghai, and provide recommendations for possible amendments in new school lunch standards of Shanghai. Twenty schools were included in the school lunch menu survey. Of those, seven schools enrolled 5389 students and conducted physical measurement of plate waste and a questionnaire survey. The amount of food and nutrients was compared according to the new China National Dietary Guideline for School Children (2016) and Chinese Dietary Reference Intakes (2013). The provision of livestock and poultry meat in menus was almost 5–8 times the recommended amount. The amount of seafood was less than the recommended amount, and mostly came from half-processed food. The average percentage of energy from fat was more than 30% in students of all grades. The greatest amount of food wasted was vegetables with 53%, 42%, and 31%, respectively, among primary, middle and high school students. Intake of Vitamin A, Vitamin B_2_, calcium, and iron was about 50% of the recommended proportion. Only 24.0% students were satisfied with the taste of school lunches. Higher proportions of livestock and poultry meat and low intake of vegetables have become integral problems in school lunch programs. Additionally, more attention needs to be paid to the serving size in primary schools with five age groups.

## 1. Introduction

Children and adolescents are in a crucial period of body growth and maturation. Adequate nutrition during this period is of great importance. A number of studies has revealed that inappropriate nutrition in childhood is related to both the occurrence of diseases in youth [1] and the risks of developing obesity, cardiovascular diseases, and cancer in adulthood [2,3,4].

Lunch becomes a very important issue when it comes to school-aged children since a large number of students have lunch at school. The National School Lunch Program (NSLP) in the United States operates in more than 101,000 public and nonprofit private schools, and provides over 28 million low-cost or free lunches to children on a typical school day [5]. In Japan, more than 10 million schoolchildren in 32,400 schools participate in the lunch program [6]. China launched the first School Lunch Program in Hangzhou, Zhejiang Province in 1987 [7], and then expanded it to a number of cities. Shanghai, as a developed city in China, started the program in 1993, and now has more than 95% students having lunch at school [8], which is a total of 1.4 million students according to Shanghai Statistic Yearbook (2016) [9].

Since lunch is correlated to the health of young generations and involves so many students, it has drawn much attention worldwide. In developed countries, such as the US and Japan, they have called a legislative action to ensure well implemented school lunch programs. The U.S. signed the National School Lunch Act (NSLA) in 1946 and the Child Nutrition Act (CAN) in 1966, as well as subsequent amendments to the two acts that guide the program’s administration [5]. Japan also introduced the School Lunch Act in 1954 and revised it in 2008 to change its aim to promote Shokuiku, which emphasizes food education. These acts clearly demonstrate the daily food and nutrient reference intake for each age group so that schools and companies that prepare lunches are able to provide adequate nutrition to students. China released the Amount of Nutritional Provision for School Lunch (ANPSL-1998) as the national standard for school lunches in 1998; there have been no amendments to date [10].

However, the health status and dietary structure of Chinese people have undergone tremendous changes within the last 20 years. Excessive intake of meat and insufficient consumption of dairy products and vegetables have emerged as concerns in Chinese dining habits [11]. Hence, it is very urgent and necessary to draw up an updated and feasible standard for the school lunch program, which requires on in-depth evaluation of the available data on the present status of school lunches. The former evaluation studies were all based on the ANPSL-1998, which might be inaccurate since China has published the new China National Dietary Guideline for School Children (2016) (CNDG-Children 2016) [12] and the Chinese Dietary Reference Intakes (2013) (DRIs-2013) [13]. To the best of our knowledge, this is the first study that applied the new guideline and DRIs to assess lunch intake in China. Additionally, unlike in the existing studies, we separated elementary, middle, and high school students into several sub-groups to obtain more information, as recommended by a recently published study by the NSLP in the US [14]. This is also the first time a comprehensive and regional-level investigation of school lunch program that involves multiple districts all over Shanghai has been conducted. As the study team of the new school lunch standardization commission in Shanghai, we aim to provide more evidence and scientific recommendations for updates.

Therefore, the objectives of this study were to evaluate the intake of food and nutrients among primary, middle and high schools students in Shanghai in reference to the CNDG-Children 2016 and the DRIs-2013 and to provide recommendations for possible amendments on new school lunch standards of Shanghai.

## 2. Methods

### 2.1. Sample Selection

Eight primary schools, five middle schools, and five high schools, two combined middle-high schools, making up a total of 20 schools, from seven districts of Shanghai, participated in the three-day lunch menu survey in 2015. Simultaneously, the intake survey and questionnaire survey were conducted among 5389 students from three primary schools (2936 students), three middle schools (1841 students), and one high school (612 students). The study population consisted of 2765 boys (51.3%) and 2624 girls (48.7%).

Signed informed consents were obtained from the students as well as their parents. No experiment and biological sample collection were conducted in this study. At no time were individual students associated with any particular lunch, and the questionnaire was anonymous without any personal identifying information, except for the students’ grade and sex.

### 2.2. Data Collection

#### 2.2.1. Menu Survey

Menu survey was a very common and easy way to assess the provision of school lunch [15,16]. Three-day lunch menus were obtained from 20 schools. Menus were analyzed with nutrient analysis software Fei Hua (2.a), Beijing Bowenshixun Technology Ltd., Beijing, China, which provided daily averages for staple food (including rice, noodles, and other cereal food), livestock and poultry meat, egg, seafood, bean products, and vegetables, as well as energy, protein, fat, carbohydrates, vitamins, and minerals. The oil, salt, and sugar used were also considered.

#### 2.2.2. Intake Survey

In seven schools, menu survey was also applied. And plate waste measures were conducted over three days, using a previously validated physical measurement of aggregate selective plate waste [17,18]. Two randomly selected sample of school lunch trays were taken from each age group serving line before lunch. Meanwhile, the weights of food before and after cooking were recorded to calculate the raw/cooked ratio (Ration of r/c). Separate trash bins with plastic bags were prepared for each food category before lunch. The research team members waited at the designated spots and separated the leftovers into the corresponding trash bin when the students finished eating and brought their trays. The number of students who had lunch at school of every day was recorded.

Food Intake per Student (cooked) in grams for each food category was calculated as follows (1):

(1) $$\mathrm{Food}\;\mathrm{Intake}\;\mathrm{per}\;\mathrm{student}\left(\mathrm{cooked}\right)\;=\;\mathrm{Sample}\;\mathrm{Weight}\;-\;\frac{\mathrm{Total}\;\mathrm{Plate}\;\mathrm{Waste}}{\mathrm{Number}\;\mathrm{of}\;\mathrm{students}}$$

Since the nutrient analysis software and the reference standard both require raw food data, the Food Intake per Student (cooked) was transformed with the raw/cooked ratio (Ration of r/c) as shown in Formula (2) below.

(2) Food Intake per student(raw) = Food Intake per student(cooked) × Ration of r/c

Plate waste is defined as the quantity percentage of edible food served as part of the lunch but not consumed, as shown in Formula (3) below.

(3) $$\%\mathrm{Plate}\;\mathrm{Waste}\;=\frac{\mathrm{Total}\;\mathrm{Plate}\;\mathrm{waste}/\mathrm{Number}\;\mathrm{of}\;\mathrm{students}}{\mathrm{Sample}\;\mathrm{weight}}\;\times \;100$$

Both menu and intake evaluation were based on the CNDG-Children 2016 for food category and the DRIs-2013 for nutrient assessment. To evaluate the lunch and nutrition intake, 40% of daily recommended intake in the CNDG-Children 2016 and the DRIs-2013 was used, as the CNDG 2016 distributes daily energy and nutrients into breakfast, lunch and dinner at the ratio 3:4:3 [19].

#### 2.2.3. Questionnaire Survey

The aim of the questionnaire survey was to find out the reason for plate waste and the existing problems in the School Lunch Program.

Validity: Three nutrition and survey research experts were invited to evaluate the appropriateness of the survey questions and response options; then, face-to-face interviews were conducted with 36 students (three students from each grade level ranging from Grades 1–12) as a pilot. The students were asked to talk about the clarity of each question to ensure the targeted respondents understand what each question is asking as well as what each response means.

The questionnaire included the following information: (1) demographic background (i.e., age, sex); (2) knowledge and attitudes: basic knowledge of food and nutrients (including six questions, one point for each correct answer, six points in total), attitude towards healthy behaviors (including four behaviors, two points for very positive attitude, one point for positive attitude and zero point for neutral or negative attitude, eight points in total); and (3) opinions on food served at the school canteen (i.e., appearance, flavor, temperature, and portion size of the food served).

Reliability: Internal consistency reliability was calculated using Cronbach’s alpha coefficient formula. Reliability of the questions related to knowledge, attitude and views on the school lunches were thus calculated to be 0.866, 0.807, and 0.792, respectively. This suggests that the reliability of this survey was adequate, since α ≥ 0.7 is generally considered to be the minimum for adequate internal consistency [20].

### 2.3. Data Analysis

Excel 2010 (Microsoft Corporation, Redmond, WA, USA) was used to record and calculate the food provided and intake. The nutrient analysis software Fei Hua (2.a) (Beijing Bowenshixun Technology Ltd., Beijing, China) was used to analyze nutrient contents. EpiData 3.1 (A comprehensive tool for validated entry and documentation of data. EpiData Association, Odense, Denmark) was used for double recording of questionnaire data. Data that presented abnormal distribution were analyzed using two independent samples via Wilcoxon rank sum test, while categorical variables were analyzed via chi-Square test using SPSS 21.0 (IBM SPSS Inc., Chicago, IL, USA). Differences found were determined to be statistically significant at *p* ≤ 0.05.

## 3. Results

### 3.1. Evaluation of Food Provision

Amounts of staple food, livestock, and poultry meat in menus exceeded the recommended amount in all school grades; livestock and poultry meat were particularly high, almost 5–8 times the recommended amounts. On the contrary, the provision of seafood was insufficient. Additionally, we found the seafood supplied for lunch was mostly fish ball or other half-processed foods, instead of fresh products. Egg provision in the diet was higher than recommended for primary and middle school students. The amount of bean products and vegetables was generally adequate for all grades (see Table 1).

Energy indicated in the menus was aligned with the recommendations. However, the proportion of fat in total energy exceeded the recommended percentage (20–30%). Protein was served excessively, at nearly twice the recommended amount, especially among primary school students. The percentage of energy from carbohydrate, which is supposed to be 50–65%, was found to be below the lower limit. As for the vitamins and minerals, Vitamin B_2_ and calcium were insufficient. Vitamin A, Vitamin B_1_, Vitamin C, iron, and zinc contents were consistent with the goals specified in the DRIs-2013 for students in all grades (see Table 2).

### 3.2. Evaluation of Food Intake and Plate Waste

The intake of staple food for grades 1–2 and middle school students was insufficient (with plate waste ranging from 15–24%), but was adequate for grades 3–5 and high school students (with 16–21% of plate waste). All students consumed livestock and poultry meat in amounts exceeding the recommended amount. Egg intake was in accordance with the recommended amounts, except among high school students. Seafood intake was in a severe shortage for primary school and high school students since their provision was also insufficient. Bean product consumption was deficient among primary school students, but reasonable among middle and high school students. Vegetable consumption was the lowest and the accompanying plate waste levels ranked the highest, with 53%, 42%, and 31%, of waste among primary, middle, and high school students, respectively. Livestock and poultry meat, and egg followed as the second ranking plate waste among all food categories (see Table 3). Additionally, students of lower grades in primary school seemed to waste more food than students of higher grades (*p* < 0.05). Girls tended to waste more food and nutrients than boys (*p* < 0.05).

Energy intake for students in Grades 1–9 was under the recommended amount. The intakes of protein and fat for all students were excessive, while the carbohydrate intake was deficient. Primary school students were facing a lack of vitamins in their diet, including Vitamin B_1_, Vitamin B_2_, and Vitamin C. Calcium intake was very low for all students. Iron intake was low except for boys in high school. Zinc intake was matched with the recommended amount. In summary, Vitamin A, Vitamin B_2_, calcium, and iron were the most deficient micronutrients in school lunches, which corresponded to about 50% of the recommendation (see Table 4).

### 3.3. Analysis for Possible Reasons for Plate Waste

A total of 5937 students from seven schools were administered questionnaires, of which 5389 (90.8%) valid responses were included in this study.

Results from the questionnaire showed that the percentage of students who often or always had leftovers was 56.3%. The main reason for plate waste among primary school students was food being too much (43.3%) and unpalatable food (43.4%). However, the main reason for plate waste in 54.5% of the middle school and 61.9% of the high school students was unpalatable food.

As for knowledge about nutrition, students from primary, middle, and high schools scored 4.44 ± 1.50, 4.69 ± 1.21 and 4.92 ± 0.98, respectively, with accuracy over 70%. However, their scores towards healthy behaviors were relatively low, at 5.80 ± 2.10, 4.25 ± 2.46, and 3.15 ± 2.18, for primary, middle, and high school students, respectively.

Students rated the food temperature and portion size as satisfactory; however, they were not satisfied with food appearance and flavor. Only 24% of the students marked the overall food taste as good (see Table 5).

## 4. Discussion

The latest report on nutrition status and chronic diseases of Chinese people issued in 2015 revealed that the wasting percentage of Chinese adolescents aged 6–17 years was 9%, while the overweight and obesity percentages were 9.6% and 6.4%, respectively [21]. Diet-related problems including anorexia and obesity have been increasing among school children [6]. School lunch, as one of the important meals in a day, also provides direct access to nutrition for students.

Nevertheless, problems continue to exist in the School Lunch Program. In the present study, we evaluated the provision and actual intake, as well as the students’ opinion on school lunches to uncover the existing problems and come up with feasible recommendations when amending the new school lunch standards of Shanghai. In particular, the provision of livestock and poultry meat was too excessive while seafood was in a severe shortage. This study highlighted the fact that excessive provision of animal protein in school lunch diets may be associated with a greater intake of fat. This high-level of fat content in school lunches is a common issue worldwide. To decrease children’s access to lunches with a high fat content, the US Department of Agriculture implemented School Meals Initiative for Healthy Children in 1995 [22]. However, this program did not show favorable results, since the three School Nutrition Dietary Assessment Studies showed that the average percentages of energy from total fat were 38% (school year 1991–1992), 33–34% (school year 1998–1999) and 33.8% (school year 2004–2005), respectively [23,24,25]. The higher-fat provision in school lunch should be given more attention as obesity has become a global public health threat [26].

The actual food intake was also unsatisfying. The plate waste of livestock and poultry meat, eggs and vegetables was higher than other food category. The provision of livestock and poultry meat, as well as eggs, far exceeded the recommended amounts, and this may be the reason for high plate waste. However, the situation for vegetables was different. The vegetable provision was within the recommended range, but accounted for the highest plate waste. Having further investigated, we found that vegetables were prepared in a large cauldron, in a manner that resulted in overcooking. Additionally, the time between cooking and consumption was about 1.5–2 h, and individual lunch sets were covered with a plastic covering to prevent contamination. This may have resulted in the vegetables gaining an unpleasant color and unpalatable flavor, leading to increased plate waste.

In fact, vegetables were reported to be the food items that are wasted the most, and this is very common all over the world [18,27]. A study involving students from grades 3–8 in four schools in the U.S. showed the waste percentage of vegetables was up to 58.9% [28]. Another study conducted in Beijing, China also indicated that vegetables were the dominant wasted food with 42% of plate waste [29], comparable with the present study ranging from 31–53%.

Due to high plate waste of vegetables and other foods, intake for energy and a majority of nutrients did not meet the recommended targets in this study. The reason for higher intake of fat and protein was the unreasonable high fat content of lunches. Intake of micronutrients, such as Vitamin A, Vitamin B_2_ and iron, was also less than the recommended level, which might be a consequence of high plate waste, especially for vegetables. However, we should notice that, for most Chinese people, they are used to eat more at dinner rather than lunch. Hence, the distribution of breakfast, lunch and dinner at the ratio 3:4:3 might not be the occasion and whether the nutrient intake over a full day is adequate remains unknown. Calcium insufficiency was also found in this study. As people usually drink milk in the morning or at night, it is difficult to judge the intake of calcium at lunch. However, the insufficiency of calcium intake has always been a problem among Chinese students because of low-milk dietary habits of Chinese people [21].

A number of factors could influence food intake, causing the unreasonable nutrient intake among students. Previous studies have concluded that students’ knowledge, attitude, and eating behaviors [29,30], as well as characteristics of the food itself (including the appearance, flavor, and temperature) [31], were the main influencing factors leading to plate waste. It was inspiring that the students achieved about 70% accuracy when answering nutrition-related questions. However, their attitudes toward healthy behaviors were not very positive. Therefore, future nutrition education should focus more on how to encourage students to turn their good nutrition knowledge into actions. In terms of the characteristics of food, the results revealed a low satisfaction in the appearance of food (19.8%) and flavor (28.1%), which might be a result of comprehensive factors, such as cooking skills of the kitchen staff, food quality, food preparation equipment and storage and so on. Furthermore, 50.4% students were satisfied with the food temperature, showing that the supply chain worked quite successfully.

Plate waste may also be due to serving size. We found that younger students wasted more food than the older ones in primary school. This must be addressed since the lunch patterns and serving sizes for food were similar within school level (primary, middle, and high school). For example, a primary school may contain five grades of different age students, but their serving sizes are the same. In addition, the recommendations for different age groups do not correspond with the actual real-life situation. DRIs-2013 for school children and CNDG-Children 2016 determined the recommendations at three age levels, i.e., 7–10 years, 11–13 years, and 14–17 years. However, the primary, middle, and high schools in Shanghai include students aged from 7–11 years, 12–15 years, and 16–18 years old, respectively. Apparently, the age group in DRIs-2013 and CNDG-Children is different from the actual situation. It should be mentioned that ANPSL-1998 successfully matched the age groups with actual school stage and separated recommendations for two age groups for primary school (i.e., students aged 6–8 years and 9–11 years). However, the recommendation for students older than 15 years is absent in ANPSL-1998. Hence, some gaps between the present recommendations and the actual situation were found. Whether the younger students in primary school need less energy, nutrients, or smaller portion sizes remains unknown. This is in accordance with the opinion of Niaki et al. from the U.S. [14]. Another study from Portugal and Denmark also suggested that portion sizes need to be reconsidered in School Lunch Program [32,33].

The physical measurement of plate waste was recommended and commonly used in dietary surveys in China [17]. Its advantages of providing detailed and accurate plate waste information were also demonstrated in a report to the U.S. Congress [18]. It overcomes the need to rely on students’ memory or lack of ability to accurately estimate portion sizes, which are common limitations of 24 h recall investigations [34]. However, the measurement of plate waste in this study was an average estimation based on total plate waste across food items rather than the individual plate waste. We did not take into account any differences in individual behaviors. Measurement of individual plate waste is quite costly and time-consuming, especially for samples over 50–100 persons [35]. Hence, visual estimation and digital photography methods have been applied in some plate waste studies [36,37,38]. Digital photography was proven to be a more accurate method to estimate plate waste since it can be standardized and offers a way to enhance the reliability and validity of recording dietary intake [36,38]. The digital photography method is worth further development, although it is not used widely in China yet.

This study was conducted in Shanghai so it has limitations to generalize to other cities or at the national level. However, some findings, such as excessive provision of livestock and poultry meat, low intake of vegetables and low satisfaction about school lunches from students, were very common across China.

## 5. Conclusions

Based the above, the recommendations for the new school lunch standards of Shanghai are as follows: (1) emphasize the provision of less livestock and poultry meat, and more fresh seafood instead of half-processed products; (2) recommend mixed-vegetable dishes. Leafy vegetables could be cooked with other food categories, such as bean products and mushrooms to improve the flavor of vegetables. More importantly, multi-component interventions should be encouraged, which was proven to be an effective way to increase the consumption of vegetables in many countries [6,39,40]; (3) recommend yogurt at lunch or milk at breakfast but this is not compulsory according to Chinese dietary habits; and (4) supplement the recommendations for students aged 15–18 years to cover the missing points in the old standard (ANPSL-1998), while separating the recommendations for primary school students as in the old standard.

## Figures and Tables

**Table 1:** Provision of food in the menus of twenty schools as compared to CNDG-Children 2016/g.

Category	Primary School		Middle School		High School	
	Recommended	Menu	Recommended	Menu	Recommended	Menu
Staple Food	**60**–**80**	99.6 ± 31.8	**90**–**100**	104.7 ± 42.7	**100**–**120**	154.3 ± 60.4
Livestock & Poultry Meat	**16**	123.4 ± 38.4	**20**	101.3 ± 44.9	**20**–**30**	135.4 ± 51.6
Egg	**10**–**16**	26.0 ± 27.8	**16**–**20**	22.8 ± 25.8	**20**	15.1 ± 20.6
Seafood	**16**	11.4 ± 23.7	**20**	28.3 ± 49.4	**20**–**30**	13.2 ± 24.5
Bean Product	**6**	9.0 ± 17.0	**6**	15.8 ± 25.5	**6**–**10**	32.2 ± 35.6
Vegetable	**120**	130.6 ± 60.8	**160**–**180**	189.2 ± 63.7	**180**–**200**	178.4 ± 84.5

Recommended amount is 40% of daily recommended intake in China National Dietary Guideline for School Children (2016) (CNDG-Children 2016).

**Table 2:** Provision of energy and nutrients in 20 schools as compared to DRIs-2013/*n* (%).

Item	Primary School		Middle School		High School	
	DRIs	Menu	DRIs	Menu	DRIs	Menu
Energy (kcal)	621	696 ± 139	783	752 ± 229	844	1005 ± 179
		(112.0)		(96.0)		(119.1)
Protein (g)	17.4	35.9 ± 7.5	23.4	32.0 ± 7.9	25.3	41.8 ± 10.0
		(206.3)		(136.8)		(165.2)
Protein/%E	-	21 ± 4	-	18 ± 5	-	17 ± 4
Fat/%E	20–30	32 ± 10	20–30	39 ± 10	20–30	37 ± 12
Carbonhydrate/%E	50–65	47 ± 10	50–65	43 ± 7	50–65	46 ± 12
Vitamin A (μgRE)	199	204 ± 184	258	258 ± 173	272	221 ± 144
		(102.5)		(100)		(81.3)
Vitamin B_1_ (mg)	0.39	0.48 ± 0.21	0.5	0.49 ± 0.20	0.54	0.60 ± 0.21
		(123.1)		(98.0)		(111.1)
Vitamin B_2_ (mg)	0.39	0.33 ± 0.09	0.48	0.32 ± 0.08	0.51	0.37 ± 0.09
		(84.6)		(66.7)		(72.5)
Vitamin C (mg)	26	39 ± 29	36	60 ± 34	38	54 ± 32
		(150.0)		(166.7)		(142.1)
Calcium (mg)	390	140 ± 85	413	217 ± 196	375	226 ± 183
		(35.9)		(52.5)		(60.3)
Iron (mg)	5.14	5.65 ± 1.77	6.28	6.91 ± 4.71	6.38	8.50 ± 4.71
		(109.9)		(110.0)		(133.2)
Zinc (mg)	2.81	4.42 ± 1.65	3.66	4.28 ± 1.25	3.75	5.52 ± 1.52
		(157.3)		(116.9)		(147.2)

DRIs are 40% of average of daily recommended intake in different age and sex group according to Chinese Dietary Reference Intakes (2013) (DRIs-2013). The value in brackets was the proportion that provision amount took up in the correspondent recommendation.

**Table 3:** Actual food intake and plate waste percentage in seven schools among different age groups and sexes/g (%).

Stage		Staple Food	Livestock & Poultry Meat	Egg	Seafood	Bean Product	Vegetable
Primary School	Recommended	60–80	16	10–16	16	6	120
	Menu	79.5	141.6	24.9	0.4	5.0	101.6
	Grades 1–2	52.9	73.7	10.5	0.2	2.4	38.9
		(34%)	(46%)	(50%)	(50%)	(50%)	(57%)
	Grades 3–5	63.1	75.0	14.8	0.2	3.2	45.1
		(21%)	(45%)	(30%)	(50%)	(33%)	(50%)
	Average Intake	58.8	74.5	13.0	0.2	2.9	42.5
		(27%)	(46%)	(38%)	(50%)	(40%)	(53%)
Middle School	Recommended	90–100	20	16–20	20	6	160–180
	Menu	93.4	119.4	34.9	27.9	14.9	176.7
	Grades 6–7 (Male)	74.5	106.3	16.2	25.8	10.5	118.2
		(11%)	(14%)	(29%)	(12%)	(20%)	(35%)
	Grades 6–7 (Female)	64.4	96.7	14.1	21.7	7.9	104.1
		(23%)	(22%)	(38%)	(26%)	(40%)	(43%)
	Grades 8–9 (Male)	81.0	95.1	16.3	25.6	9.0	105.3
		(3%)	(23%)	(28%)	(13%)	(32%)	(42%)
	Grades 8–9 (Female)	61.5	82.1	15.2	24.9	8.8	87.8
		(26%)	(34%)	(33%)	(15%)	(33%)	(52%)
	Average Intake	70.9	96.7	15.5	24.6	9.2	105.9
		(15%)	(22%)	(32%)	(16%)	(30%)	(42%)
High School	Recommended	100–120	20–30	20	20–30	6–10	180–200
	Menu	140.4	164.3	18.4	2.0	23.0	198.1
	Grades 10–12 (Male)	149.0	161.0	17.6	2.0	18.6	154.2
		(1%)	(0%)	(24%)	(0%)	(18%)	(27%)
	Grades 10–12 (Female)	103.0	141.1	13.4	1.3	14.4	139.4
		(31%)	(12%)	(42%)	(35%)	(37%)	(34%)
	Average Intake	126.7	151.3	15.5	1.6	16.6	147.0
		(16%)	(6%)	(33%)	(20%)	(27%)	(31%)

**Table 4:** Actual intake and proportion to the recommended amounts of energy and nutrients in seven schools among different age groups and sexes/*n* (%).

Stage		Energy	Protein	Fat	Carbonhydrate	Vitamin A	Vitamin B_1_	Vitamin B_2_	Vitamin C	Calcium	Iron	Zinc
		kcal	g	%E	%E	μgRE	mg	mg	mg	mg	mg	mg
Primary School	Recommended	662	19	20–30	50–65	212	0.42	0.42	28	416	5.5	3.0
	Menu	638	35	35	42	156	0.38	0.32	28	95	4.7	4.3
		(96.4)	(188.2)			(73.6)	(91.3)	(76.9)	(100.0)	(22.8)	(85.4)	(143.3)
	Grades 1–2	409	20	37	43	58	0.19	0.19	10	48	2.9	2.5
		(61.8)	(107.5)			(27.4)	(45.7)	(45.7)	(35.7)	(11.5)	(52.2)	(83.3)
	Grades 3–5	480	22	38	43	79	0.25	0.21	12	60	3.1	2.8
		(72.5)	(118.3)			(37.3)	(60.1)	(50.5)	(42.9)	(14.4)	(56.6)	(93.3)
	Average Intake	451	21	37	43	70	0.23	0.20	11	55	3.0	2.7
		(68.1)	(112.9)			(33.0)	(55.3)	(48.1)	(39.3)	(13.2)	(54.7)	(90.0)
Middle School	Recommended	835	25	20–30	50–65	275	0.53	0.51	38	440	6.7	3.9
	Menu	665	32	38	43	232	0.59	0.30	55	168	5.1	3.8
		(79.6)	(128.0)			(84.4)	(111.3)	(58.8)	(144.7)	(38.2)	(76.1)	(97.4)
	Grades 6–7 (Male)	612	29	42	39	148	0.63	0.25	35	117	4.0	3.2
		(73.3)	(116.0)			(53.8)	(118.9)	(49.0)	(92.1)	(26.6)	(59.6)	(82.1)
	Grades 6–7 (Female)	542	26	43	38	127	0.60	0.23	30	104	3.5	2.9
		(64.9)	(104.0)			(46.2)	(113.2)	(45.1)	(78.9)	(23.6)	(52.8)	(74.4)
	Grades 8–9 (Male)	599	28	40	41	131	0.58	0.24	32	105	3.8	3.2
		(71.7)	(112.0)			(47.6)	(109.4)	(47.1)	(84.2)	(23.9)	(56.7)	(82.1)
	Grades 8–9 (Female)	499	24	42	39	112	0.48	0.20	26	89	3.2	2.6
		(59.8)	(96.0)			(40.7)	(90.6)	(39.2)	(68.4)	(20.2)	(48.1)	(66.7)
	Average Intake	570	27	42	39	132	0.58	0.23	31	106	3.7	3.0
		(68.3)	(108.0)			(48.0)	(109.4)	(45.1)	(81.6)	(24.1)	(55.2)	(76.9)
High School	Recommended	900	27	20–30	50–65	290	0.58	0.54	40	400	6.8	4.0
	Menu	924	46	35	46	242	0.71	0.40	61	180	7.5	5.4
		(102.7)	(170.4)			(83.4)	(121.9)	(74.1)	(152.5)	(45.0)	(110.3)	(135.0)
	Grades 10–12 (Male)	910	45	30	51	190	0.70	0.39	45	146	6.9	5.3
		(101.1)	(166.7)			(65.5)	(120.7)	(72.2)	(112.5)	(36.5)	(101.5)	(132.5)
	Grades 10–12 (Female)	697	36	33	46	158	0.55	0.31	40	112	5.0	4.1
		(77.4)	(133.3)			(54.5)	(94.8)	(57.4)	(100.0)	(28.0)	(73.5)	(102.5)
	Average Intake	807	40	32	49	175	0.62	0.35	43	129	6.0	4.7
		(89.7)	(148.1)			(60.3)	(106.9)	(64.8)	(107.5)	(32.3)	(88.2)	(117.5)

The value in brackets was the proportion that actual intake took up in the correspondent recommendation.

**Table 5:** Students’ opinion on school lunches/*n* (%).

Rank	Good	Neutral	Bad
Appearance	1067 (19.8%)	2689 (49.9%)	1633 (30.3%)
Flavor	1515 (28.1%)	2705 (50.2%)	1169 (21.7%)
Temperature	2716 (50.4%)	2177 (40.4%)	496 (9.2%)
Adequate in Portion Size	2792 (51.8%)	1918 (35.6%)	679 (12.6%)
Overall Food Taste	1293 (24.0%)	2905 (53.9%)	1191 (22.1%)
